# Sonography of the distal urethra in lambs

**DOI:** 10.1186/s13028-017-0283-2

**Published:** 2017-03-14

**Authors:** Ammar AlLugami, Kerstin von Pückler, Axel Wehrend, Marlene Sickinger

**Affiliations:** 1 Clinic for Obstetrics, Gynecology and Andrology of Large and Small Animals with Ambulatory Services, Faculty of Veterinary Medicine, Justus-Liebig-University Giessen, Frankfurter Str. 106, 35392 Giessen, Germany; 2 Clinic for Small Animals, Department of Surgery, Faculty of Veterinary Medicine, Justus-Liebig-University Giessen, Frankfurter Str. 108, 35392 Giessen, Germany

**Keywords:** Sonography, Urethra, Urolithiasis, Small ruminants, Lambs

## Abstract

Sonography is a convenient, non-invasive diagnostic modality in small ruminants, often used in reproductive management, internal medicine, and surgery. Pregnancy diagnostics and imaging anatomy and pathology of organs, such as neoplasia, are major applications. Urolithiasis is one of the most common causes of death in male sheep and goats, for which sonography is the diagnostic modality of choice. Although ultrasound-imaging techniques for kidneys, ureters, and urinary bladder in small ruminants have been described previously, this study focuses on reporting the imaging technique of the extra-pelvic portion of the urethra, as nearly all the cases of obstructive urolithiasis result from urinary stones in this part. Thirty-three Lacaune-crossbred lambs were examined using a 12 MHz linear probe in laterally recumbent animals. Using this technique, the urethral lumen could be visualised through its entire course in all lambs.

## Findings

Presence of urinary stones (urolithiasis) is a well-known condition [1] of which the incidence has increased in humans as well as in small and large animals [2]. In small ruminants, the prevalence of urolithiasis may reach up to 10% in fattening lambs, and mortality rates of up to 100% in cases of obstructive urolithiasis are not uncommon [3]. Apart from the history and clinical examination, several diagnostic tools might be useful in diagnosing this disorder. The method of choice for confirmatory diagnosis is transabdominal sonography [4, 5]. This diagnostic modality enables the practitioner to not only validate the diagnosis but also evaluate the prognosis and determine appropriate therapeutic measures within a few minutes [4]. Besides the initial orientating abdominal sonography, further examinations, for example of the kidneys and the urinary bladder, are necessary to evaluate the findings and determine the best available surgical technique to treat obstructive urolithiasis [6]. Previous studies have provided descriptive reference values for the appearance, position, and sizes of the upper urinary tract, including the kidney, bladder, and proximal urethra. However, similar reference data for the lower urinary tract are not available. The aim of this study was to describe the findings of sonographic examination of the male ovine urethra distal to ischial arch.

Thirty-three healthy 4-month-old Lacaune crossbred fattening lambs were examined with permission of the responsible local animal welfare authority. The group of lambs consisted of castrated (n = 11) and intact lambs (n = 22) that were housed in stables with straw and received concentrates, hay, and water ad libitum. Castration was performed at the age of 3–4 weeks. Data concerning body weights, crown rump lengths (CRL) and withers heights (WH) are given in Table 1.

**Table 1:** Descriptive data for all lambs at the ages of 3 weeks and 4 months, respectively

Age	Mean body weight (kg)	SD	Mean CRL (cm)	SD	Mean WH (cm)	SD
3 weeks (n = 33)	10.8	2.7	64.5	4.8	46.7	2.8
4 months (n = 33)	32.9	4.3	90.4	5.4	62.4	3.8
Differences	22.1	1.6	25.9	0.6	15.7	1.0
Mean daily gain	0.223	0.02	0.26	0.006	0.16	0.01

All lambs were weighed and CRL as well as the WH were recorded regularly. Daily gains were calculated based on a 99 day time period

*SD* standard deviation

All examinations were performed in awake animals in lateral recumbency. The urethra was examined through its entire course using a 12 MHz linear sonography probe (Aplio XG, Toshiba). Hair was clipped and ultrasound contact gel was applied on the skin and probe for optimal contact. During the examination, attention was paid to apply only mild and uniform pressure on the probe. Sagittal and transverse imaging of the urethra was performed and verified by colour and power Doppler examinations. First, the urethra was localised by placing the probe at a 90° angle on the skin in the inguinal region to provide transverse view of the penis. Then, the probe was slowly turned counter clock-wise to provide longitudinal view of the urethra along its course. The examinations included the tip of the penis (Position 1), penile urethra (Position 2), the distal (Position 3) and proximal (Position 4) sigmoid flexure and the ischial flexure (Position 5).

Using a 12 MHz linear sonography probe, the urethral lumen could be visualised in all the intact and castrated lambs at all the five locations. Visualisation of urethra was difficult in very young rams (age around 3 weeks) or in animals that had been castrated at an early age (<4 weeks of age). Positioning in lateral recumbency was well tolerated by all the animals, showing only a minimal defensive behaviour. Tympany, arising from oesophageal obstruction to eructation, was not noticed consequent to lateral recumbency. The duration of examination was 12–30 min per animal.

**Fig. 1:**
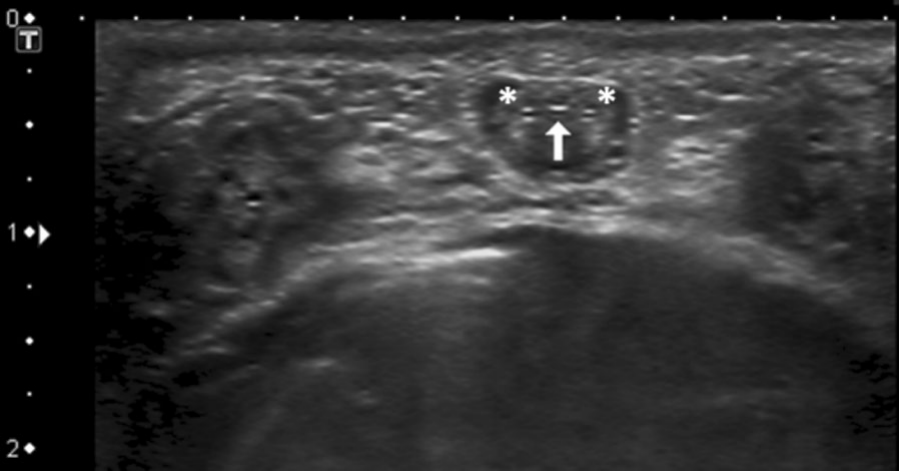
Transverse sonography of the penis of a 4-week-old ram. The urethra is flanked by two blood vessels allowing the examiner to orientate and find the urethra. *Arrow* transverse section of the urethra; *asterisk* flanking blood vessels

**Fig. 2:**
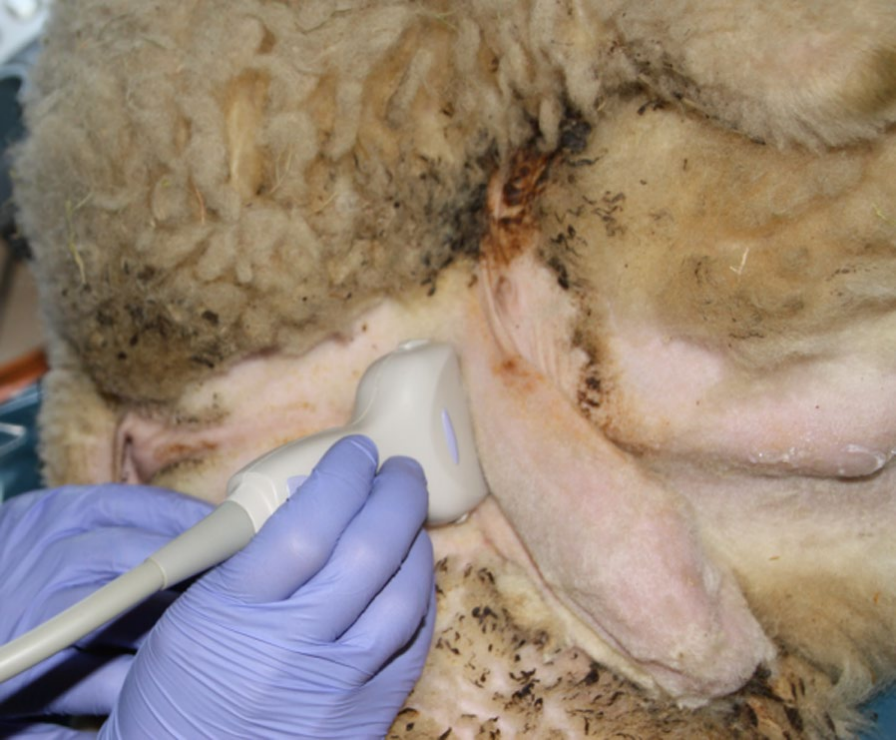
Position of the animal for examination. The ram is softly fixed in *left* recumbence. The probe position for imaging the distal sigmoid flexure in transverse section is shown

**Fig. 3:**
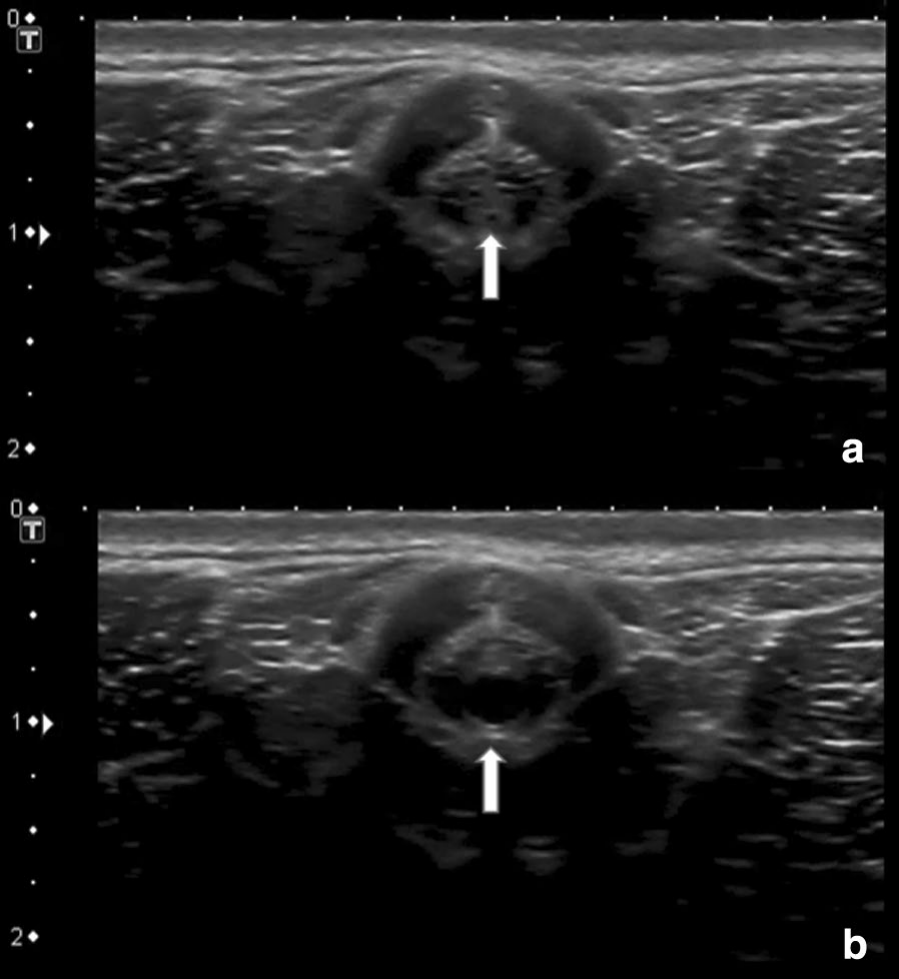
Transverse sections of the urethra during voiding. The urethra is maximally distended during voiding and collapses after suspending of the urinary outflow. **a** Transverse sonographic view of a collapsed urethra in a 4-week-old ram; **b** maximally dilated urethra of the same ram during voiding. *Arrow*: urethra

On ultrasonography the urethra appeared as an anechoic region flanked by the hyperechoic surfaces of the urethral mucosa. A triad comprising of the urethra and two collateral blood vessels facilitated orientation and location of the urethra (Fig. 1). This triad was easily recognized, especially at the site of the distal sigmoidal flexure. In order to visualize the urethra at this site, the probe was positioned at the base of the scrotum and pointed craniodorsally (Fig. 2). This technique could be applied to both castrated and intact animals. Examination of the urethra revealed that both intact and castrated males could rotate the penis within the prepuce around the longitudinal axis. Therefore, the position of urethra and the flanking blood vessels varied between ventral and lateral within the penis. The diameter of the urethral lumen varied significantly from 0.4 mm when being empty to 3 mm during spontaneous urination (Fig. 3). A marked difference between the urethral lumen in very young lambs (3–4 weeks of age) and lambs at the age of 4 months was observed. In castrated lambs at the age of 4 months, the urethral lumen was slightly smaller than in the intact lambs. Detailed data are given in Table 2.

**Table 2:** Mean diameters (in mm) and standard deviations (SD) of the urethra of intact and castrated lambs at different locations (Positions 1–5; see the text for definitions) at the age of 3 weeks and 4 months

Age/castration status	Pos. 1	Pos. 2	Pos. 3	Pos. 4	Pos. 5
3 weeks, all lambs (n = 33)	0.41	0.40	0.44	0.41	0.39
SD	0.08	0.10	0.12	0.10	0.07
4 months, all lambs (n = 33)	0.50	0.47	0.49	0.44	0.48
SD	0.14	0.12	0.12	0.10	0.13
4 months, intact (n = 22)	0.54	0.51	0.52	0.47	0.51
SD	0.14	0.10	0.12	0.10	0.13
4 months, castrated (n = 11)	0.43	0.38	0.43	0.37	0.41
SD	0.09	0.09	0.10	0.06	0.10

The lambs were castrated at the age of 3–4 weeks

In this study, the anatomy of the urethra distal to the ischial arch was examined by ultrasonography in healthy intact and castrated lambs. In contrast to the results of other studies [6–8], we could examine and visualise the urethra successfully in all animals. In small animal medical practice, the urethral lumen is often visualised only when presented with a pathologic dilatation of the urinary bladder [7, 8]. Braun et al. [6] presumably failed to show the urethral lumen in healthy rams and in cases of obstructive urolithiasis because of technical aspects of the devices used. They used 5 MHz frequency probes to image the urinary tract of 20 healthy rams and seven rams with obstructive urolithiasis. In contrast, we used a linear probe of 12 MHz frequency; higher frequency waves penetrate to lesser depth compared to the lower frequency waves. Imaging of the urethra needs only small penetration depth, as it lies superficially under the skin. In small animals, the urethra is best visualised by placing the animal in dorsal recumbency using sonography probes of 7.5 MHz or higher [9]. In ruminants, however, dorsal recumbence may obstruct eructation [10]; therefore, we performed sonography with high frequency probes, like in small animals, but in lateral recumbency.

Two other techniques used to image the urethra by ultrasound are retrograde insertion of a urinary catheter into the urethral lumen and by instillation of agitated sodium chloride solution [9]. Both techniques are used to create contrast through the introduction of microbubbles present in the catheter or the NaCl solution [11]. In this study, these techniques were not used because of the different anatomy of small ruminants’ urethra; in small ruminants, the narrow lumen of the *processus urethralis* at the tip of the penis makes the insertion of a urinary catheter difficult. The lumen of this appendix may only be reliably passed with a catheter when the filiform appendix itself has been amputated [12]. Instillation of a NaCl solution however is possible via a permanent venous catheter without stiletto. However, to do so, the patient has to be positioned in an upright sitting posture and the penis advanced out from the prepuce. In this posture, the sigmoid flexure tends to straighten and the lumen of the urethra may vary. Instilling of a NaCl solution into the urethra may cause further dilation. However, in this study, we aimed to image the urethra in its normal size and from; therefore, these techniques were avoided.

In human medicine, the two techniques used for ultrasonographic examination of urethral course and lumen in males are either by using a Foley balloon catheter inserted into the urethra and secured by inflating the balloon at *fossa navicularis* and or by examination during active urination [13]. These techniques cannot be applied directly to small ruminants due to the aforementioned presence of filiform appendix, which makes insertion of catheter difficult, and the animals cannot be commanded to urinate at will. Nevertheless, even in small ruminants, a detailed examination, enabled by urinary catheters may be of great benefit in the preparation and planning of a surgical intervention. These examinations, however, are recommended to be performed under general anaesthesia preceding the surgical procedure.

In conclusion, this study shows that the urethra in small ruminants can be consistently evaluated through its course using high frequency ultrasonographic probes. As the urethra collapses after voiding but still is visible using the described technique, the visualisation of the urethral lumen in patients with obstructive urolithiasis is presumed due to the dilatation of the urethra proximal to the obstruction in such cases. Therefore, sonography might enable detection of the cause and site of urinary obstruction through full course examination of the urethra. The non-invasive character of sonography favours its use as the method of choice in rams with voiding disturbances.
